# Extra-Uterine Pregnancy

**Published:** 1912-05

**Authors:** Floyd W. McRae

**Affiliations:** Atlanta, Ga.


### Journal-Record of Medicine

Successor to Atlanta Medical and Surgical Journal, Established 1855
and Southern Medical Record, Established 1870.

**OWNED BY THE ATLANTA MEDICAL JOURNAL CO.**

**Published Monthly**

*Official Organ Fulton County Medical Society, State Examining Board, Presbyterian Hospital, Atlanta, Birmingham and Atlantic Railroad Surgeons' Association, Chattahoochee Valley Medical and Surgical Association, Etc.*

EDGAR G. BALLENGER., M. D., Editor.
BERNARD WOLFF, M. D., Supervising Editor.
A. W. STIRLING, M. D., C. M., D. P. H., J. S. HURT, B. Ph., M. D.
GEO. M. NILES, M. D., W. J. LOVE, M. D., (Ala.); Associate Editors.
E. W. ALLEN, Business Manager.

COLLABORATORS

Dr. W. F. WESTMORLAND, General Surgery.
F. W. McRAE, M. D., Abdominal Surgery.
H. F. HARRIS, M. D., Pathology and Bacteriology.
E. B. BLOCK, M. D., Diseases of the Nervous System.
MICHAEL HOKE, M. D., Orthopedic Surgery.
CYRUS W. STRICKLER, M. D., Legal Medicine and Medical Legislation.
E. C. DAVIS, A. B., M. D., Obstetrics.
E. G. JONES, A. B., M. D., Gynecology.
R. T. DORSEY, Jr., B. S. M. D., Medicine.
L. M. GAINES, A. B., M. D., Internal Medicine.
GEO. C. MIZELL, M. D., Diseases of the Stomach and Intestines.
L. B. CLARKE, M. D., Pediatrics.
EDGAR PAULIN, M. D., Opsonic Medicine.
THEODORE TOEPEL, M. D., Mechano Therapy.
R. R. DALY, M. D., Medical Society.
A. W. STIRLING, M. D., etc., Diseases of the Eye, Ear, Nose and Throat.
BERNARD WOLFF, M. D., Diseases of the Skin.
E. G. BALLENGER, M. D., Diseases of the Genito-Urinary Organs.

VOL. LIX. MAY 1912 NO. 2

### EXTRA-UTERINE PREGNANCY.

BY FLOYD W. McRAE, M. D., ATLANTA, GA.

In choosing the subject of Extra-Uterine Pregnancy, I have been influenced largely by the fact that the majority of cases of extra-uterine pregnancy I have operated on have been referred to me as cases of appendicitis. Having done no obstetric work for a number of years, it is natural that I should not have seen as many cases of extra-uterine pregnancy as would fall in the hands of obstetric surgeons. It is also quite probable that I have seen a larger proportion of cases that have been mistaken for appendicitis, on account of the fact that I have done more general surgery, and have had a larger number of cases of appendicitis referred to me than perhaps any other one surgical disease.

The fact, however, that these cases are frequently diagnosed appendicitis is quite evident. In many particulars the symptoms are the same, but the symptoms in the main are sufficiently distinctive, if carefully gone into, to enable one to make a correct diagnosis in all but exceptional cases.

The cases of extra-uterine pregnancy that I have had referred to me with a diagnosis of appendicitis or appendiceal abscess and have operated upon, have given histories subsequently that corresponded with the pathology found at operation. If these histories had been gone into intelligently and carefully considered, they should have led to correct diagnoses.

Both diseases are characterized by sudden onset with extreme pain. Nausea and vomiting are much less marked in extra uterine pregnancy than in fulminant appendicitis. The shock is more pronounced and progressive in extra-uterine pregnancy than in appendicitis. There is less gaseous distention in extra-uterine pregnancy than in appendicitis. There is also markedly less tenderness and muscular spasm in extra-uterine pregnancy than in appendicitis. A leucocyte count is of great differential value.

The histories of the two conditions differ essentially. Vaginal and rectal examinations should enable one to arrive at a correct diagnosis where the examination is made early and in detail.

Douglas, "Surgical Diseases of the Abdomen," says: "It would appear that the history and physical signs of the accompanying menstrual disorder would distinguish a ruptured ectopic gestation from appendicitis. In both there may be amenorrhoea; in both violent pain attends the onset; in both muscular rigidity may be present, and shock is a constant symptom. In three instances, as previously stated, I have seen an attack of appendicitis bring on uterine hemorrhage. In one of these cases there was amenorrhoea for eight weeks, then hemorrhage was profuse. Vaginal examination was negative, yet an erroneous diagnosis of extra-urterine pregnancy was made. The appendix was found gangrenous and entirely separated from the caecum and an opening as large as a dime marked its former site of attachment; through this opening inestinal contents had abundantly escaped into the peritoneal cavity. The patient was submitted to operation in a little less than six hours after the occurrence of the first symptom, yet there was evidence of general peritonitis."

This paper, dealing only with early ectopic pregnancies, is intended to be both an exposition and a confession—an exposition of the mistakes of others, a confession of my own mistakes. It is needless in this company of doctors for me to say how much more familiar I am with my own mistakes, how much less lenient I should be with them than with the mistakes of others.

While a very large proportion of ectopic pregnancies occur in multiparae, just one-half of my cases were primaparae, two of them unmarried, presumably virgins. One of them a school girl only seventeen.

The first case of extra-uterine pregnancy that I operated on many years ago, was referred to me by the late Dr. Stockard, and was operated on at his Sanatorium, located on Walton St. The patient was a primipara and had been a morphine habitue for years. The case had been diagnosed by Dr. Stockard as appendicitis, and I confirmed the diagnosis. I found a very large abscess resulting from an infected ruptured right tubal pregnancy. The clots were removed, the abscessed cavity wiped out and copious drainage left. The patient made a very slow recovery. The drainage of this abscess left a persistent sinus which I cleared out eighteen months later, removing the appendix, which evidently had nothing to do with the original pathology.

My next case was a young woman 28 years old with cancer of the cervix. I had submitted a piece of tissue from the cervix to Dr. Harris, from which he made a positive diagnosis of cancer. There had been slight menstrual irregularity, but nothing very suspicious. There was a small mass in the right side of the pelvis which I took to be a hydro-salpinx, but which proved to be a ruptured tubal pregnancy which was removed, together with the uterus per vaginam. This patient made an uninterrupted recovery, has had no recurrence of the cancer and is well to-day. This was in 1900—twelve years since operation.

My next case was Mrs. W.—age 19, whom I saw in consultation at College Park in what seemed to be an attack of appendicitis. There had also been a diagnosis of pregnancy; she had persistent nausea and also a slight enlargement in the appendiceal region apparently not connected with the uterus. The latter organ was enlarged and felt like a pregnant uterus. The patient was moved to the Piedmont Sanatorium. The vomiting persisted to such a degree that it was deemed absolutely necessary to empty the uterus. So I proceeded to do a curettment; found nothing but decidua in the uterus. The patient's condition was extreme, but I felt that it was my duty to investigate the right side. This proved to be an ovarian pregnancy of between two and three months' duration. The right ovary was a bluish tumor the size of an orange. Patient made an uneventful recovery.

The next case, Miss A., age 17, a schoolgirl, though having a history of not having menstruated for ninety days, was above suspicion in the mind of her doctor. He made a diagnosis of fulminant appendicitis. When I saw her, there was a large mass in the right side and evidence of infection. Without making a vaginal examination, I concurred in the diagnosis of appendicitis. The operated disclosed an infected ruptured tubal pregnancy. The young lady made a good recovery, is at home with her family and no one the wiser except those in immediate attendance.

I have only had one case of repeated ruptures. Mrs. S—, age 35 whose history is extremely interesting. The first rupture took place about three weeks before the operation at her home in Mississippi; shock and signs of hemorrhage pronounced. Dr. Smythe, of Memphis, Tenn., was called in and made a correct diagnosis, but advised that her condition was too extreme to admit of surgery. She improved some, and two weeks later came to Hogansville, Ga., on the train, where she improved steadily until the day of operation, July 11, 1907. Early in the morning she was seized with sudden violent pain, extreme shock and depression. A pelvic tumor which was easily palpated by her father, who was a physician, increased rapidly in size; patient cold and clammy; pulse weak and rapid, sighing respiration, subnormal temperature. Operation, about three o'clock in the afternoon, looked to be utterly without hope when we commenced, while patient was being infused. On opening the abdomen a large quantity of blood gushed out. I removed a quantity of foetal and placental tissue, together with broken-down clots; drainage left in, through and through sutures. Patient's condition extreme for several days, but finally made a good recovery.

These cases, which were operated on rather at haphazard and without careful diagnoses, taught me a number of very important lessons, and have enabled me, together with a most careful study of the subject and thorough investigation, to make correct diagnoses in the following cases:

Mrs. J., age—, referred by attending physician with diagnosis of appendiceal abscess. Ruptured tubal pregnancy of several weeks' standing; infection of the mass; general septic condition. Abscess cavity quickly cleaned out, drainage. Death from exhaustion a few hours after operation. Patient in extremis when put on the table.

Mrs. L.—age 26. About one month before operation, August 18, 1910, had congestion of the bowels with pain in right ovarian region, and a great deal of nervousness. When pain came on was ten days past time for period. At first had slight menstrual discharges; during the last two weeks quite free. Great deal of backache and pain in lower part of abdomen. Diagnosis—Fallopian pregnancy. Operation through McBurney incision. Right ovary as large as a lime and practically destroyed; tube a dark color and about the size of a hen's egg. Tube and ovary removed. Appendix adherent at the tip of the tube—appendectomy. Uninterrupted recovery.

Miss __________, young unmarried woman about 23 years old; diagnosis of appendicitis by attending physician. History of sudden onset, shock, pain in the right side several days before; lower abdomen considerably distended. In making a pelvic examination, a large soft mass was found occupying the whole right side of the pelvis and extending well above the brim. Vagina patulous and dilatable; gave history of irregular menstruation during past three or four months, containing more or less membranous debris. I made a tentative diagnosis of ruptured ectopic pregnancy. On opening the abdomen, found a large, dark tumor which confirmed the diagnosis of extra-uterine pregnancy. Ruptured right tube, clots and membranes removed; drainage left. Patient made a somewhat tedious but good recovery.

Mrs. S.—Age 32. Child seven years old. Menstruation irregular—periods from three to six weeks, at one time during past year missing four months; no signs of pregnancy. On November 15, 1910, had a sudden, severe pain in right side. On the following day, while on the train from Nashville, had a second attack of pain. Got off at Atlanta and went to the Piedmont Sanatorium. Operation November 22. Found a large blue tumor about the size of a baby's head, involving the right tube and ovary, and just on the point of second rupture. Removed and patient made good recovery.

Mrs. H.—aged 32, was seen by one of Atlanta's best obstetric and abdominal surgeons the day before I was called in, and a diagnosis of fulminant appendicitis was made. Patient had missed one menstrual period. About two weeks before admission to the Infirmary, June 10, 1911, pain began in right ovarian region and continued steadily growing worse. Had slight flow. On going into her history and making a careful examination, I made a diagnosis of probable ruptured tubal pregnancy. After watching her for four days, I decided positively that the trouble was ectopic and on operation found this to be true. There were large quantities of dark blood in the pelvic cavity and a large distended right tube with a small perforation. The tube was removed, leaving the ovary which seemed perfectly normal. Recovery.

Mrs. C—age 32. Mother of three children. Last child seven years old. On June 19, 1910, had a trachelorrhaphy and perineorrophy done. Health perfect since operation; periods had been regular. Menstruation began 16th of May, 1911, and continued to operation. A week previous, began having severe colicy pains in left side. Operation June 16, 1911. Quite a mass in left side. Found abdomen filled with old clotted blood and ruptured left tubal pregnancy. Tube removed and cystic ovary resected. Uterus large, friable, edematous, so that partial hysterectomy was deemed advisable. Prompt recovery.

Mrs. A., age 35, is the wife of a college professor of science who has written out in detail one of the most classical histories of ectopic pregnancy that I have ever read. It is well worth any doctor's perusal. This case, while presenting a clear history, was seen by four physicians, two of them amongst the best gynecologists and abdominal surgeons in Atlanta, and a diagnosis of appendicitis was made by all of them. Extra-uterine pregnancy was discussed, but rather dismissed by a process of elimination. When I saw the patient, after getting a most lucid history from her husband, I felt quite sure that she had ruptured tubal pregnancy and that the mass in the right side was ectopic. On examination at a later day, I confirmed this opinion, which was proven correct on the operating table.

"Mrs. A. was born August 21, 1875. In the spring of 1891, she suffered a severe attack of typhoid fever; the fever ran high and the course of the disease normal. In February, 1895, she was taken sick with severe cramps and abdominal pains, and in a few days she was suffering from an acute attack of typical peritonitis. The attack was very severe. The crisis was passed on Easter night of that year. Her recovery was slow and tedious and it was evident that the disease had left its mark within, as she was noticeably lame in the right leg and suffered for months with acute pain the right hip and in the region of the right ovary.

On June 6, 1896, she was married to Mr. A., then a student at Johns Hopkins University, Baltimore, Md. At this time her menstrual periods were quite irregular and were accompanied with much pain. The lameness in the right leg had almost disappeared, but the movement of this leg was sluggish, causing a slight halt in her step. Her right shoulder was carried slightly forward, causing a marked stoop. The pain in right hip had disappeared except at menses, at which time it was often a fierce, throbbing pain, hard to endure. Her condition remained about the same until it was thought advisable to consult Dr. Howard Kelly, of Baltimore, in the spring of 1898 (this may have been in late fall of 1897.) Dr. Kelly examined her and reported very serious adhesions on the right side involving the ovary, tube and surrounding parts, but because of good general health, advised letting nature take care of conditions until a more urgent call for an operation came. He advised outdoor exercise and freedom from worry.

Four years later, after she had moved to ———————— Ga. her condition was not improved. In fact, the pain in region of the ovary, the delayed menstruation, the stoop in walking and at times very annoying leucorrhoea, made another consultation advisable. This time Dr. Noble examined her and confirmed Dr. Kelly's statements concerning the adhesions, but expressed the opinion that she could never be a well woman without an opration and suggested that she might grow worse at any time. He advised an operation, but expressed serious doubts about pregnancy after such an operation, and in other ways made the operation appear so serious that it was indefinitely postponed. This was in 1902. A few months later, in hope of improving local uterine conditions, opening up womb and possibly making menstruation easier, she submitted to a curettment.

During the summer of 1905 she became pregnant. By the end of the first month she was suffering extremely from nausea, which continued for three months. This, with stoppage of menstruation, and distinct signs in the breasts, made it very evident that she was pregnant. She came to term in good physical condition and on April 5, a fine boy, 10½ lbs., was delivered with a little assistance of the attending physician, Dr. Ernest. Within two months her health in every way was much improved and when menstruation began a year later, it was regular and painless and has been so ever since. In the fall of 1907, she became pregnant again, and in the second or third month of pregnancy, an abortion was brought on by undertaking to lift a heavy piece of furniture. The flow which resulted continued for six weeks, but she was in bed only two or three days, and was soon in good health again. In September, 1909, she gave birth to a daughter, the labor being perfectly normal. After confinement she came back to her usual good health and remained so until the next fall, when she went to bed with a slow fever that seemed like a slight typhoid or a paratyphoid fever. She had recovered and up to and during the summer of 1911, she was enjoying the best health of her life. She had been regular in menstruation until August. She missed this period and when the third week was passed, thought she was pregnant. On September 12, while en route from W. Va. to Georgia, she was greatly frightened because she thought her little boy would be run over by the train. The next morning a show of blood appeared and she thought it was her delayed menstrual flow, but it continued for only a part of the day, instead of three days as usual, almost disappeared, and then in a few days appeared again. This, accommpanied by considerable pain in the right side and general debility. These conditions continued for about two weeks and the discomfort and pain increased until there were attacks of cramps in the abdomen. Two marked attacks of cramps had occurred within the next week, when suddenly she was seized with the most intense pain, with rigors. and cramps, and Dr. Sweet, of Agnes Scott College, was called in. She administered morphine and tried to relieve her with turpentine stupes. Her condition was little improved over night, and Dr. Sawyer was called, in and on examination the womb was found much enlarged, but the tenderness was so great that the examination could not proceed far. Her bowels were evacuated with epsom salts and an enema and turpentine stupes continued. There was considerable distention and pain from gas, and the tenderness of the whole abdomen was marked. During the day the tenderness became more localized in region of the appendix. Her temperature was running normal throughout most of the day, but at night it would run up between 100 and 101, which continued for several days, her general condition improving. On the fifth day without any warning she suddenly passed into another rigor with most intense pain, a very fast and wiry pulse and extreme weakness, accompanied with a profuse cold sweat. There was great accumulation of gas and some evidence of obstruction to its passage, so she was given a heavy dose of castor oil and two enemas. The enemas brought little return. Three hours later the castor oil acted freely, bringing together with much mucus, the first seen during the sickness, a firm mass of clear mucus as large as a golf ball, imbedded with grape seeds and fully a teacupful of loose grape seeds. She had eaten freely of grapes eight days before, had had at least two natural movements, two movements from epsom salts and two enemas during this period with no signs of grapes. A movement the next day brought out another mass of thick mucus with abundant grape seeds. After this her condition improved rapidly and she was soon sitting up. She remained quite weak and the flow continued, soon settling down to a rather intermittent flow, free one day and then letting up for six or eight days. Her severe attack came Sunday night, October 8th. On Monday, October 20, she was examined by Drs. Sawyer, McRae and Sweet, and a distinct mass was located in the right side, the nature of which was hard to determine. Two weeks later she was again examined by Dr. McRae, and it was found that the mass had evidently developed and, considering the history, he pronounced it extra-uterine pregnancy, and on December 5th, she was taken to the Piedmont Sanatorium. On December 6th, she was operated on by Dr. McRae and his diagnosis of tubal pregnancy fully confirmed. The tube had ruptured, probably at the late severe attack. It was also seen that the cause of the peritonitis in 1895 was due to an attack of appendicitis, during which the appendix had ruptured and had been almost completely obliterated."

A study of the above twelve histories, only one of which is given in detail, discloses several interesting facts. All but one were right-sided; six were diagnosed appendicitis; one was diagnosed hydro-salpinx; five correctly diagnosed before operation; eleven recoveries and one death. The mistakes in diagnosis were not hurtful to the patient, but were rather humiliating to the operator. The attending physicians are to be commended rather than criticized for urging prompt operations in conditions so evidently surgical and demanding early interference. It was up to the operating surgeon to differentiate, to make proper diagnoses.

In conclusion, please allow me to again quote extensively from "Surgical Disease of the Abdomen."—Douglas.

"Clinical Picture before Rupture.—Ectopic pregnancy prior to rupture has been repeatedly recognized and successfuly treated surgically. One operator, Dr. Baldwin, of Columbus, Ohio, has reported eleven such cases. It has not fallen to my lot to make this early diagnosis.

When a patient in comparatively good health, with a previous history of amenorrhoea varying from a few days to two or three weeks, or else of protracted menstruation with continued spotting, and complaining of vague atypical, not necessarily colicy pains in the pelvis, of peculiar sensations, morning nausea, and sore breasts, we should think of the possibility of ectopic gestation. The pelvic examination, showing a slightly enlarged uterus, a small, movable, elastic, pulsating and sensitive tubal tumor, lying laterally to or behind the uterus, the contiguous ovary separately palpable, and the opposite adnexa healthy, we have sufficient evidence to warrant a provisional diagnosis of intact ectopic gestation.

If the pains become more conspicuous and of a more definitely colicy character, if uterine hemorrhage is more profuse and contains decidual membrane, if the tumor is larger than the probable stage of gestation warrants, if such tumor is oblong, sausage-shaped, extremely sensitive, hard and tense, the inference is that there has been intra-tubal bleeding, that gestation is at an end, and that a tubal mole has formed.

When a patient with the above history suffers with severe colicy pains, or with pain of an entirely different character, sharp and cutting, attended by slight attacks of syncope, which show marked tendency to recur at varying intervals; and when upon local examination we detect a boggy mass behind the uterus, the presumption is that upon operation we shall find intraperitoneal hematocele, due to partial or complete tubal abortion."

"Clinical Picture at the Time of Rupture.—The symptoms of primary intra-peritoneal rupture are those of intra-peritoneal hemorrhage, characterized by severe stabbing, lancinating, agonizing pains, felt in the lower portion of the abdomen, sometimes quite definitely upon one side, occasionally quite remotely referred, accompanied by shock, usually somewhat prolonged, rarely deepening into profound collapse; further evidenced by acute anaemia, with its rapid fickering pulse, ashy-pale face, blanched mucous membranes, cold extremities and low temperature; by hurried respiration, thirst, perhaps nausea and vomiting, sometimes great restlessness, or else an oblivious apathy, occasionally passing into a wild delirium and ending in death. More commonly there is a slow reaction. The patient shows the effect of the storm through which she has passed by her great protration; she complains of dizziness and "blind spells;" her bowels are constipated; frequently there is retention of urine; she also complains of soreness in her abdomen, which is found to be rigid and hypersensitive, or, if a few hours have elapsed, there is some distention, and we may be able to detect by the slow changing dulness in the dependent regions the evidences of free fluid. Vaginal examination shows a soft cervix with slightly gaping os, an enlarged uterus, behind which there is a sensation of fulness, and sometimes a definite mass can be outlined. A distinct tubal tumor may be felt if the gestation has advanced beyond six weeks. But not infrequently the pelvic examination is unsatisfactory, if not entirely negative. It is very probable that, while conducting this examination, fresh hemorrhage will be excited, the patient will complain of faintness, and her pulse will increase in frequency. These symptoms may be elicited immediately at the time of rupture, or several days may have elapsed. Usually their severity receives prompt diagnosis and treatment, or else death removes the patient from our sphere of observation."

In discussing Dr. McRae's paper, Dr. J. N. Ellis said that much of his difficulty had been in the reverse of Dr. McRae's. He had diagnosed tubal pregnancy and found appendicitis instead. He showed a specimen of inflamed appendix that had been adherent to the omentum and Wood ligament, making a tumor that resembled the pregnant tube. He related a case of tubal pregnancy that ruptured into the sigmoid and manifested the presence of extensive haemorrhage of clot and undigested blood from the rectum. Both these cases recovered after operation.

Dr. E. C. Davis said, as to the frequency of ectopic pregnancy, it occurred much more frequently than was understood. A coroner in Philadelphia found in the autopsies of women 5% were ectopic. His personal experience is from 50 to 75 cases of ectopic pregnancy, and he believes it occurred so often that more attention and thought should be given to the matter so as never to overlook a case. It is a very serious condition and should be recognized whenever arising. In the early rupture of these cases the woman rarely bleeds to death. She can be tided over and the proper operation can be done. After the 3rd or 4th month a difficult problem is opened up. Would Dr. McRae advise sequestration in these cases or continue to do the marsupialization as formerly?

Dr. McRae said he had little to do with late cases. Dr. Davis's question as to waiting for the viability of the child and being guided by that, that opened up so serious a problem that he would prefer discussing it at another time. He believes the condition is more frequent than we know. The symptoms should all be carefully marshaled and studied in every case resembling it and then the diagnosis is not difficult. He agrees that death does not generally come with the first haemorrhage.

